# Rhodobacteraceae dominate the core microbiome of the sea star *Odontaster validus* (Koehler, 1906) in two opposite geographical sectors of the Antarctic Ocean

**DOI:** 10.3389/fmicb.2023.1234725

**Published:** 2023-09-20

**Authors:** Emanuela Buschi, Antonio Dell’Anno, Michael Tangherlini, Sergio Stefanni, Marco Lo Martire, Laura Núñez-Pons, Conxita Avila, Cinzia Corinaldesi

**Affiliations:** ^1^Department of Marine Biotechnology, Stazione Zoologica di Napoli “Anton Dohrn”, Fano Marine Centre, Fano, Italy; ^2^Department of Life and Environmental Sciences, Polytechnic University of Marche, Ancona, Italy; ^3^Department of Research Infrastructures for Marine Biological Resources, Stazione Zoologica di Napoli “Anton Dohrn”, Fano Marine Centre, Fano, Italy; ^4^Department of Biology and Evolution of Marine Organisms, Stazione Zoologica di Napoli “Anton Dohrn”, Naples, Italy; ^5^Department of Integrative Marine Ecology, Stazione Zoologica di Napoli “Anton Dohrn”, Naples, Italy; ^6^NBFC, National Biodiversity Future Center, Palermo, Italy; ^7^Department of Evolutionary Biology, Ecology and Environmental Sciences, Faculty of Biology, University of Barcelona, Barcelona, Catalonia, Spain; ^8^Institut de Recerca de la Biodiversitat, University of Barcelona, Barcelona, Catalonia, Spain; ^9^Department of Materials, Environmental Sciences and Urban Planning, Polytechnic University of Marche, Ancona, Italy

**Keywords:** microbiome, microbial diversity, *Odontaster validus*, geographic location, Antarctica

## Abstract

Microbiota plays essential roles in the health, physiology, and in adaptation of marine multi-cellular organisms to their environment. In Antarctica, marine organisms have a wide range of unique physiological functions and adaptive strategies, useful for coping with extremely cold conditions. However, the role of microbiota associated with Antarctic organisms in such adaptive strategies is underexplored. In the present study, we investigated the diversity and putative functions of the microbiome of the sea star *Odontaster validus*, one of the main keystone species of the Antarctic benthic ecosystems. We compared the whole-body bacterial microbiome of sea stars from different sites of the Antarctic Peninsula and Ross Sea, two areas located in two opposite geographical sectors of the Antarctic continent. The taxonomic composition of *O. validus* microbiomes changed both between and within the two Antarctic sectors, suggesting that environmental and biological factors acting both at large and local scales may influence microbiome diversity. Despite this, one bacterial family (Rhodobacteraceae) was shared among all sea star individuals from the two geographical sectors, representing up to 95% of the microbial core, and suggesting a key functional role of this taxon in holobiont metabolism and well-being. In addition, the genus *Roseobacter* belonging to this family was also present in the surrounding sediment, implying a potential horizontal acquisition of dominant bacterial core taxa via host-selection processes from the environment.

## 1. Introduction

Animal–bacterial symbioses are a ubiquitous feature of marine life for many vertebrate and invertebrate taxa and are widespread among the five echinoderm classes (Zilber-Rosenberg and Rosenberg, 2008; Gilbert et al., 2012; McFall-Ngai et al., 2013; Carrier et al., 2018). Approximately, 60% of echinoderms studied so far, including sea stars, establish relationships with subcuticular bacteria (i.e., localized in the lumen between epidermal cells and the outer cuticle; Burnett and McKenzie, 1997; Lawrence et al., 2010; Høj et al., 2018), which appear to be related to host phylogeny (McKenzie et al., 1998; Høj et al., 2018).

Previous findings revealed that microbial members associated with several echinoderm species belong mainly to Alpha- and Gammaproteobacteria, and in particular to the families Phyllobacteriaceae and Rhizobiaceae, which are generally involved in nitrogen fixation, and to the order Chromatiales, involved in sulfur oxidation (Lawrence et al., 2010). Symbiotic bacteria have been reported to mediate amino-acid uptake and facilitate the supply of nutrients to their echinoderm hosts (Roberts et al., 1991; Lesser and Walker, 1992; Lawrence et al., 2010; Galac et al., 2016). In addition, they can also have an important role in the early developmental stages; and the structure of the sea star larval microbiome tends to change depending on the feeding regime (Carrier and Reitzel, 2017; Carrier et al., 2018).

Several studies have documented host species-specific associations and the presence of a core microbiome (i.e., the suite of members shared among microbial consortia from similar habitats; Shade and Handelsman, 2012; Rubio-Portillo et al., 2018), which generally remains stable even under changing environmental conditions (Dunphy et al., 2019). Nonetheless, multiple drivers including biological (e.g., feeding behavior, health status) and environmental factors (e.g., nutrient availability, geographic location) may influence simultaneously host-associated microbiota, and conflicting results have been obtained so far in attempts to disentangle the contribution of these drivers in shaping microbiomes (Pantos et al., 2015; Reese and Dunn, 2018; Schuelke et al., 2018; van de Water et al., 2018; Griffiths et al., 2019; Boscaro et al., 2022).

Antarctic ecosystems have been characterized by major events during the past million years, thus influencing genetic connectivity, producing a unique and incredibly diverse marine community, composed of around 17,000 marine invertebrate species and with the highest percentage of endemic species of any other continent (Janosik and Halanych, 2010; Chown et al., 2015; Peck, 2018). Similar to larger metazoan organisms, historical processes, such as geographic distances, dispersal barriers and oceanographic mechanisms, might substantially contribute to microbiomes’ patterns, rather than local environmental factors (i.e., habitat type, depth; Hanson et al., 2012, 2019). Recent investigations in echinoderm holobionts revealed congruency between Antarctic symbiotic microbiota compositions and corresponding sea urchin hosts, in both cases seemingly influenced by the same oceanographic and ecological factors (Schwob et al., 2021).

The sea star *Odontaster validus* Koehler, 1906 is a keystone species of Antarctic benthic communities (Dearborn, 1977; Zenteno-Devaud et al., 2022), which lives at depths down to *ca.* 900 m, and has different functional roles in food webs due to its plasticity in feeding behavior (i.e., omnivore, filter feeder, scavenger, herbivore, predator active, scavenger, spongivore; Pearse, 1969; McClintock, 1994; McClintock et al., 2008; Baum and Worm, 2009; van den Hoff et al., 2014; Zenteno-Devaud et al., 2022). Investigations on the evolutionary history of *O. validus* highlighted that the distribution of this species is confined to benthic ecosystems surrounding the Antarctic continent and islands and demonstrated a long-distance genetic similarity due to the vast dispersal potential of the pelagic larval phase lasts for 6 months or more (Janosik et al., 2011). Despite the ecological importance of *O. validus*, information on the microbiome of this sea star is very limited. So far, the only available information on the microbiome of *O. validus* and other sea star species suggests that environmental changes can trigger microbial dysbiosis (Núñez-Pons et al., 2018; Aquino et al., 2021; Loudon et al., 2023). Nevertheless, available information on microbial associations with Antarctic invertebrates suggests a functional role of the microbiome in different host metabolic processes (e.g., nutrient metabolism, and detoxification processes; González-Aravena et al., 2016; Herrera et al., 2017; Lo Giudice and Rizzo, 2018).

In the present study, we investigated the whole-body bacterial microbiome of the sea star *O. validus* inhabiting the Antarctic Peninsula and several sites of the Ross Sea, the two main marine areas located in opposite geographic sectors of the Southern Ocean. These two distinct Antarctic seas, despite being connected by the Antarctic Circumpolar Current (which has been described as a promoter of genetic connection across the sub-Antarctic zone; Moon et al., 2017; Zenteno-Devaud et al., 2022), are influenced by different oceanographic processes including the presence of large and deep-reaching cyclonic gyres (Orsi et al., 1993; Jacobs et al., 2002; Carter et al., 2008; Chown et al., 2015) and trophic conditions (Smith et al., 2012), leading to hypothesize a different influence on the sea star microbiome. To test this hypothesis, we assessed the diversity, putative functions, and potential origin (from the surrounding habitats) of the bacterial microbiome of *O. validus* from the two different Antarctic sectors.

## 2. Materials and methods

### 2.1. Study area and samples collection

Sampling was carried out during the Antarctic expedition ACTIQUIM-4 in the South Shetland Islands (Antarctic Peninsula) and during the XXXIV Italian Expedition in Antarctica at Terra Nova Bay (Ross Sea) in the framework of the Italian National Program of Antarctic Research (PNRA). Five sampling sites were selected: Port Foster’s Bay, located in the Antarctic Peninsula, and Amorphous Glacier, Punta Calizza, Spiaggetta Tethys Bay and Adelie Cove, located in the Ross Sea area (Supplementary Figure 1; Supplementary Table 1). Port Fosters’ Bay is located in the center of Deception Island in the north of the Antarctic Peninsula. It is a dynamic environment characterized by historical volcanic eruptions and strong tidal currents variations (Smith et al., 2003; Vidal et al., 2011). Amorphous Glacier, Punta Calizza, Tethys Bay and Adelie Cove are four sites of the Ross Sea area, located in the south part of Antarctica. The Ross Sea is the most productive and richest area in terms of species of the Southern Ocean (Smith et al., 2012). Due to the presence of large, deep-reaching cyclonic gyres, the Antarctic Peninsula and the Ross Sea can present diversified features and endemic species (Orsi et al., 1993; Jacobs et al., 2002; Carter et al., 2008; Chown et al., 2015). A total of 28 individuals of ~5 cm diameter of *O. validus* were collected by scuba diving at 25 m water depth. The specific number of individuals of *O. validus* collected in each site and the codes used to refer to them are shown in Supplementary Table 2. Specimens were preserved in ethanol (95%) and stored at −20°C both for taxonomic identification and microbiome characterization. Samples of surrounding sediments were collected in these sites of the Ross Sea area using plexiglass cores and stored at −20°C. Due to logistical issues with sampling equipment sediment samples could not be collected in the Antarctic Peninsula.

### 2.2. Morphological and molecular identification of *Odontaster validus*

Individuals of *O. validus* were morphologically identified through dichotomous keys (Ludwig, 1903; Koehler, 1917; Clark, 1962, 1963; Clark and Downey, 1992; Presler and Figielska, 1997; Janosik and Halanych, 2010; Janosik et al., 2011). The molecular identification of each individual was done by sequencing the coding mitochondrial 12S rDNA genes and part of the mitochondrial protein-coding COI gene (Vences et al., 2005a,b; Yang et al., 2014). Amplification of selected mitochondrial markers was performed using the primer sets: 12Slev (5′-GCCAGCAGCCGCGGTTA-3′) and 12Sdes1 (5′-CCTACTTTGTTACGACTTAT-3′; 482–505 bp; Trontelj and Utevsky, 2005), LCO1490 (5′-GGTCAACAAATCATAAAGATATTGG-3′) and HCO2198 (5′-TAAACTTCAGGGTGACCAAAAAATCA-3′; 630 bp; Folmer et al., 1994). Reaction mixtures consisted of 5 μL of 5× My Taq Reaction Buffer (Bioline), 0.5 μL of each primer (20 μM), 0.5 μL of My Taq HS DNA Polymerase (5 U/μL concentration; Bioline), 1 μL of DNA template and nuclease-free water prefiltered through a 0.02 μm pore size filter to reach a final volume of 25 μL. The thermal cycling profiles consisted of an initial denaturation of 5 min (2 min for COI gene) at 95°C, followed by 35 cycles of 30 s at 95°C (94°C for COI gene), 30 s at 50°C (12S gene) or at 48°C (COI gene), 45 s at 72°C, with a final extension of 10 min at 72°C. PCR products were verified by 1% agarose gel electrophoresis using 10.000× GelRed Nucleic Acid Stain (Biotium), 0.4 gr of agarose, 40 mL of TE Buffer for the gel preparation, and 2 μL of 5× GelPilot DNA Loading Dye (Qiagen), 2 μL of GeneRuler 1 kb DNA Ladder (Thermo Fisher Scientific) for the electrophoresis. Subsequently, they were purified using Qiagen PCR Purification Kit and sequenced using Sanger Technology (Sanger et al., 1977) and Applied Biosystems 3,730 DNA Analyzer 48 capillaries (Life Technologies). The sequences obtained were analyzed using the software Geneious 7.1.9 (Kearse et al., 2012). The terminal section of the sequence including low-quality reading and the primers were removed before assembling the two strands into consensus sequences. Both strands of all PCR products were sequenced using the same primers used for the amplification. Multiple alignments for each marker were performed using MUSCLE algorithm (Edgar, 2004) in Alivew 1.26 (Larsson, 2014). Additional sequences used as conspecific groups were downloaded from GenBank (http://www.ncbi.nlm.nih.gov/genbank/; EF624444.1 and GU227092.1, for 12S and COI, respectively) and were used to confirm the identity of our samples. Sequences were grouped in haplotypes using DNA Sequence Polymorphism (DNASP v6. 12.03) program (Rozas et al., 2017) and visualized in the phylogenetic trees of the separate 12S and COI dataset with MEGA X (Kumar et al., 2018). Sequences of *O. validus* are available in GenBank database (Bioproject ID: PRJNA984618).

### 2.3. Molecular analysis of the bacterial microbiome associated with *Odontaster validus* and sediments

DNA was extracted from a whole-body 3 mm-long section of tissue from each individual of *O. validus*, using the Qiagen DNeasy Blood and Tissue Kit (Brasier et al., 2016) and following the manufacturer’s instructions with a slight modification (i.e., extended incubation with proteinase K at 56°C overnight to better lyse the outer sea star tissues). Total DNA from the sediments was extracted using the PowerSoil DNA Isolation Kit, following a modified protocol (Danovaro, 2009): initial treatment with a set of washing solutions and 10 min of incubation at 70°C was carried out to achieve a greater extraction efficiency. The washing solutions used are WS1 (50 mM Tris–HCl, pH 8.3; 200 mM NaCl; 5 mM Na2EDTA; 0.05% Triton X-100), WS2 (50 mM Tris–HCl, pH 8.3; 200 mM NaCl; 5 mM Na2EDTA) and WS3 (10 mM Tris–HCl, pH 8.3; 0.1 mM Na2EDTA). PCR amplifications were performed on an approximately 550 bp fragment of the hypervariable V4 region of the 16S rRNA gene, using the bacterial primer set identified by Klindworth et al. (2013). The reaction mixture consisted of 37.5 μL of filtered and autoclaved Milli-Q water, 10 μL of 5x My Taq Reaction Buffer (Bioline), 0.25 μL of each primer (100 μM), 1 μL of My Taq HS DNA Polymerase (5 U/μL concentration), 1 μL of DNA extracted. The thermal cycling consisted of 2 min at 95°C, followed by 35 cycles of 30 s at 95°C, 30 s at 53°C, and 45 s at 72°C, with a final extension of 5 min at 72°C. Successful DNA amplification was verified by 1% agarose gel electrophoresis using 10.000x GelRed Nucleic Acid Stain (Biotium), 0.4 gr of agarose, 40 mL of TE Buffer for the gel preparation, and 2 μL of 5x GelPilot DNA Loading Dye (Qiagen), 2 μL of GeneRuler 1 kb DNA Ladder (Thermo Fisher Scientific) for the electrophoresis. The amplified DNA was sequenced on an Illumina MiSeq sequencer using the V3 technology (2 × 300 bp) with the same primers used for the PCR amplification at LGC Genomics. Sequences of *O. validus* and sediment microbiomes are available in the GenBank database (Bioproject ID: PRJNA984618).

### 2.4. Bioinformatic and statistical analysis

Raw sequences were analyzed through the QIIME2 pipeline (version 2019.4; https://qiime2.org/). Paired-end sequence files were loaded, and sequence pairs were analyzed using the DADA2 plugin (Callahan et al., 2016), which infers community composition in each sample by partitioning sequences according to the respective error models, thus filtering for erroneous reads and chimeras and resolving minimal variations between prokaryotic taxa; trimming parameters were set at 250 bp for forward-facing reads and 190 bp for reverse-facing reads. Paired sequences were then merged by the pipeline before producing an ASV table. Each sample was subsampled to 1,400 sequences, thus obtaining a normalized ASV table. Five individuals of *O. validus* were discarded because they were characterized by a very low number of sequences (74, 76, AG3, CAL1, CAL5). The normalized ASV table was used for the calculation of rarefaction curves and as input for the subsequent analyses, such as the determination of α and β diversity indices (Shannon and Pielou’s Evenness indices, Bray Curtis dissimilarity). To infer the taxonomic affiliation of ASVs, a taxonomic classifier was first trained on the SSU region amplified by the primers utilized in the present study on the SILVA reference database v138 and then used on the ASVs identified (Quast et al., 2012); comparisons in taxonomic composition of microbiomes were performed using the statistical packages within the STAMP program (Parks et al., 2014). To predict the relevant potential functions of microbiomes a functional annotation using FAPROTAX database (Louca et al., 2018) was done. This database maps prokaryotic taxa to putative functions using information based on functional annotations of cultivated representatives. Significant differences (*p*-values < 0.05) in the richness, taxonomic composition and putative functions of microbiomes were highlighted through a permutational analysis of variance (PERMANOVA) Similarities and dissimilarities among the different groups were evaluated by classification-clustering based on the Bray Curtis similarity of transformed data with SIMPER analysis, both included in the PRIMER-E 6 software (Anderson et al., 2008).

## 3. Results

### 3.1. Molecular identification of *Odontaster validus* individuals

Molecular analyses of the individuals of *O. validus* was performed to confirm the morphological identification and to avoid the presence of cryptic species. Primer sets used for the fragments of mitochondrial 12S successfully amplified the genomic DNA of all 24 individuals of *O. validus*, while the primer pairs used for the COI gene provided good quality PCR products in only 7 individuals. The alignment of 12S sequences and the resulting phylogenetic tree revealed that the 24 sequences of *O. validus* grouped into 2 shared and 6 unique haplotypes. Similar results were obtained with the alignment of COI sequences, where the resulting phylogenetic tree revealed that the 6 sequences of *O. validus* grouped into 1 shared and 5 unique haplotypes. Nevertheless, all the haplotypes cluster with known sequences of *O. validus* downloaded from GenBank both considering the 12S and COI genes, and identifying all the individuals under investigation as *O. validus.*

### 3.2. Microbiomes associated with *Odontaster validus*

**Figure 1:**
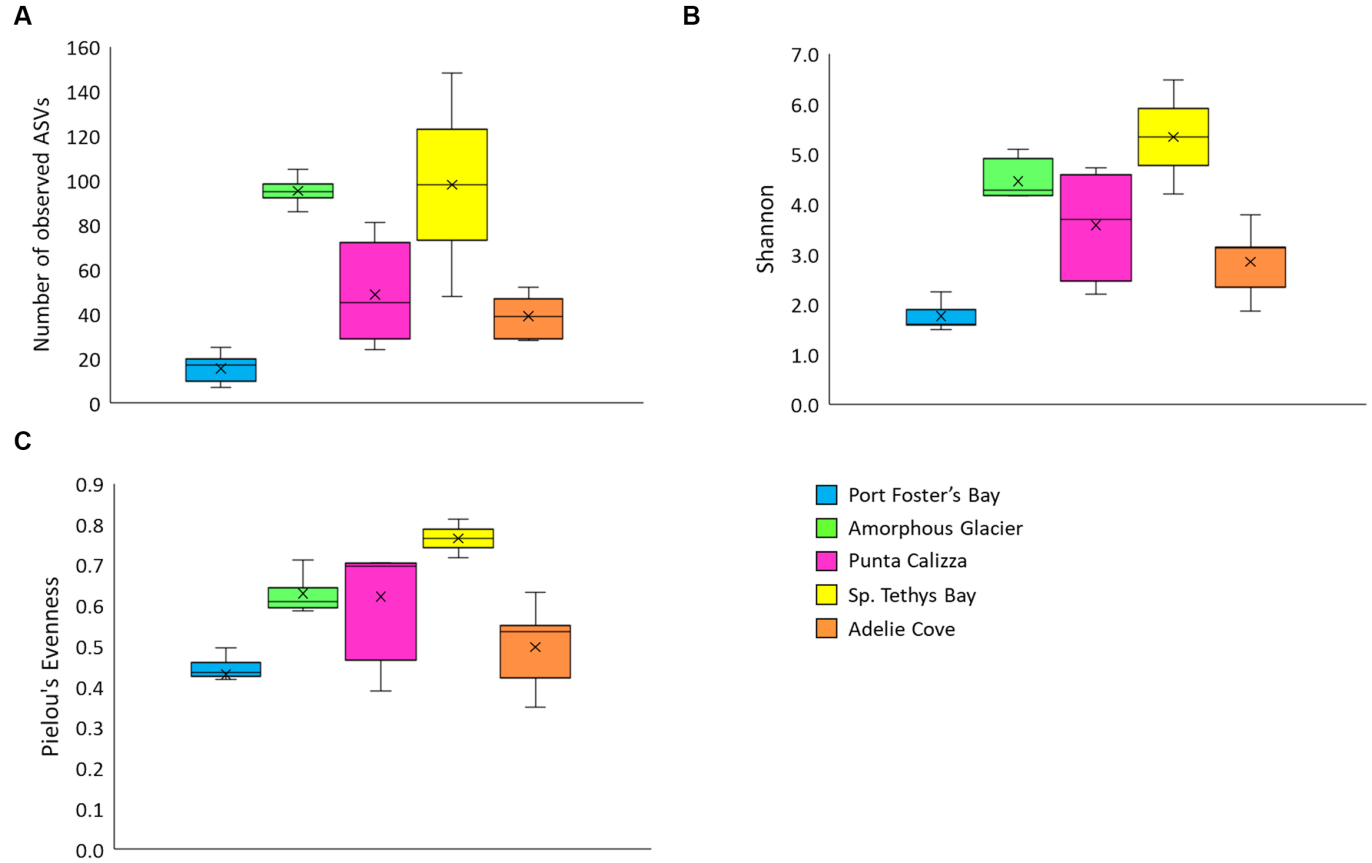
Box plots of ASV richness **(A)**, Shannon **(B)** and the Pielou’s Evenness **(C)** indices of alpha-diversity of microbiomes associated with individuals of *Odontaster validus* collected in the different Antarctic sites of the Antarctic Peninsula and Ross Sea areas. The “x” within each box plot indicates the average value.

The sequencing depth of the 16S rRNA gene exhaustively covered the bacterial microbiome diversity associated with *O. validus* individuals (Supplementary Figure 2). The number of observed ASVs varied from 7 to 25 in Port Foster’s Bay (Antarctic Peninsula) and from 86 to 105, from 24 to 81, from 48 to 148, from 28 to 52 in Amorphous Glacier, Punta Calizza, Sp. Tethys Bay and Adelie Cove (within the Ross Sea area), respectively. Significant differences in terms of ASV richness were found among individuals of *O. validus* collected in the different sites, with the lowest average number of ASVs found in Port Foster’s Bay (Antarctic Peninsula) and the highest in Sp. Tethys Bay (Ross Sea; Figure 1A; Supplementary Table 3). Similar trends were observed for the Shannon and the Evenness indices, with the lowest values in the individuals of Port Foster’s Bay, and the highest values in individuals of Sp. Tethys Bay (Figures 1B,C). Significant differences among the different Antarctic sites were also observed considering the β-diversity of microbiomes of *O. validus*, calculated on Bray Curtis dissimilarity (Supplementary Figure 3).

**Figure 2:**
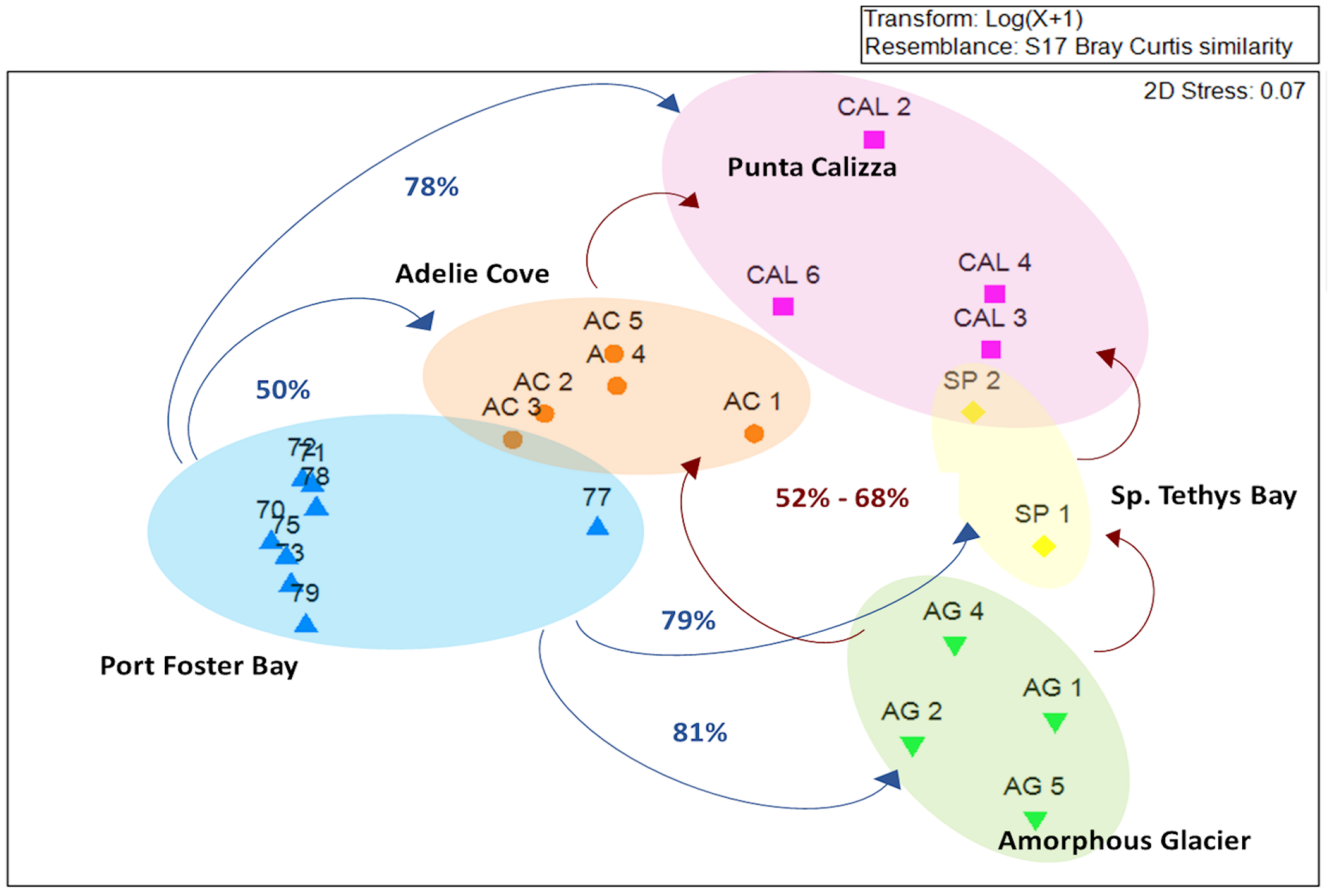
MDS analysis comparing the taxonomic composition of microbiomes associated with individuals of *Odontaster validus* collected in the different Antarctic sites of the Antarctic Peninsula and Ross Sea areas. Percentages in blue refer to the dissimilarities between Port Foster Bay and the different Ross Sea sites; percentages in red refer to the dissimilarities among the sites within the Ross Sea area.

**Figure 3:**
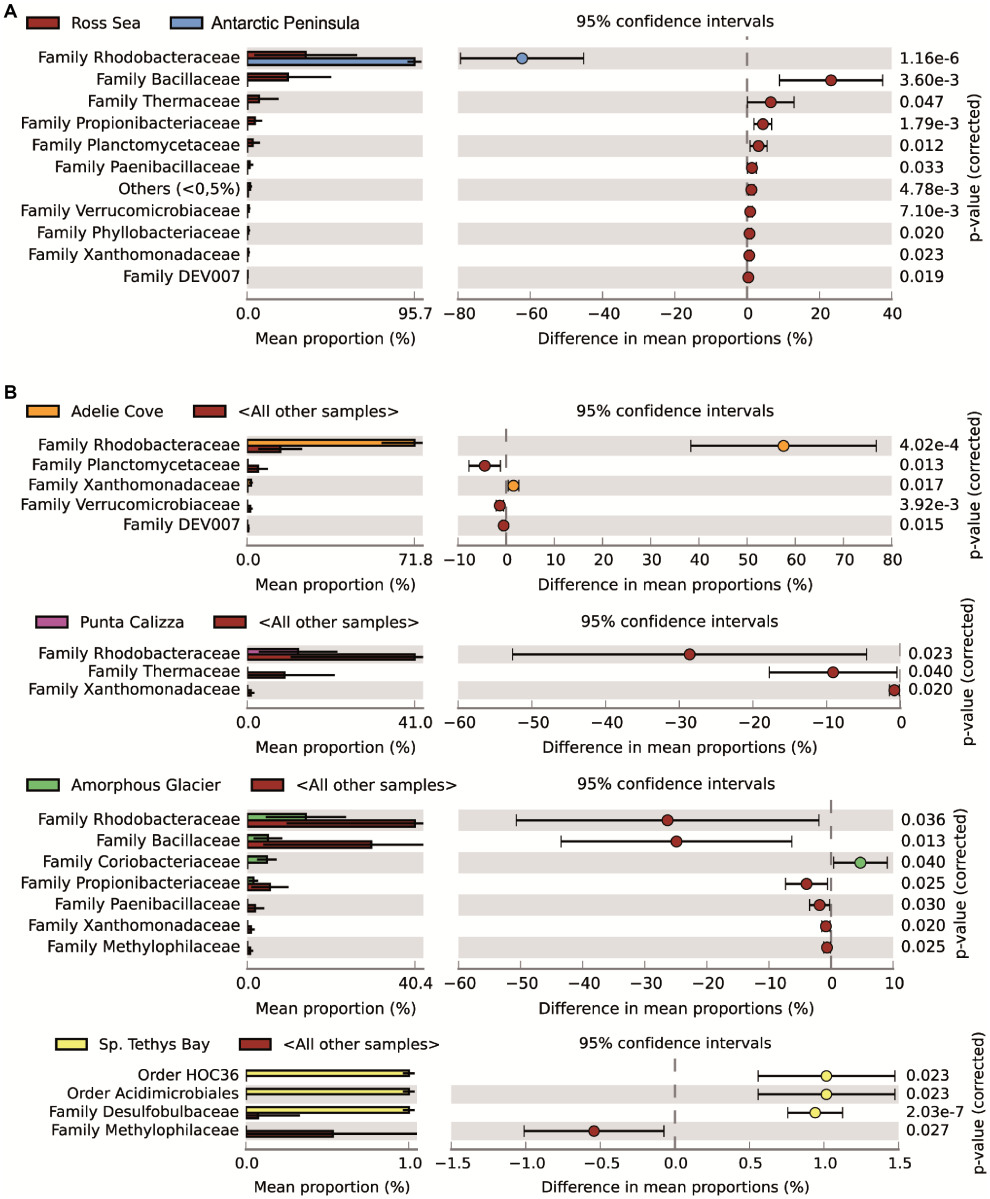
Main bacterial taxa responsible for the differences between microbiomes associated with *Odontaster validus* collected in the Antarctic Peninsula and the Ross Sea areas **(A)**. Main bacterial taxa responsible for the differences among microbiomes associated with *O. validus* collected in the different benthic sites within the Ross Sea area **(B)**. Comparisons were performed used as statistical test the White’s non-parametric *t*-test (value of *p* < 0.05).

The taxonomic annotation allowed us to identify a total of 26 different bacterial phyla of which the most abundant were α-Proteobacteria, Firmicutes, Deinococcus-Thermus and Actinobacteria with an average contribution of 55%, 21%, 5%, and 4%, respectively. Moreover, we identified 156 different bacterial families of which only Rhodobacteraceae (mainly represented by *Roseobacter* and *Sulfitobacter* genera) was shared in all individuals, with different abundances depending on the different benthic sites (on average for 95%, 14%, and 13%, 17 71% in individuals collected in Port Foster’s Bay, Amorphous Glacier, Punta Calizza, Sp. Tethys Bay and Adelie Cove, respectively; Supplementary Figure 4). Microbiomes associated with the *O. validus* collected in the Antarctic Peninsula area were characterized by a similarity in the taxonomic composition of 67%, largely explained by the Rhodobacteraceae family that represented on average 95% of the total microbiome. Microbiomes associated with the *O. validus* collected in the Ross Sea area displayed a similarity in the taxonomic composition of 45%, explained by a core that represented on average 60% of the total microbiomes and composed of 3 bacterial families: the Rhodobacteraceae, Bacillaceae and Propionibacteriaceae (on average the 55%, 38% and 7% of the core). Significant differences were found in the microbiomes of individuals of *O. validus* collected in the two sectors of Antarctica, the Antarctic Peninsula and the Ross Sea (average dissimilarity of 71%; Figure 2; Supplementary Tables 3, 4), mainly driven by the higher abundances of Rhodobacteraceae in the Antarctic Peninsula than in the Ross Sea (95% vs. 33%, respectively), and by the higher abundances of Bacillaceae, Propionibacteriaceae and Thermaceae families in Ross Sea than in the Antarctic Peninsula (on average 23%, 4%, 7% vs. below 0.2%, respectively; Figure 3A).

**Figure 4:**
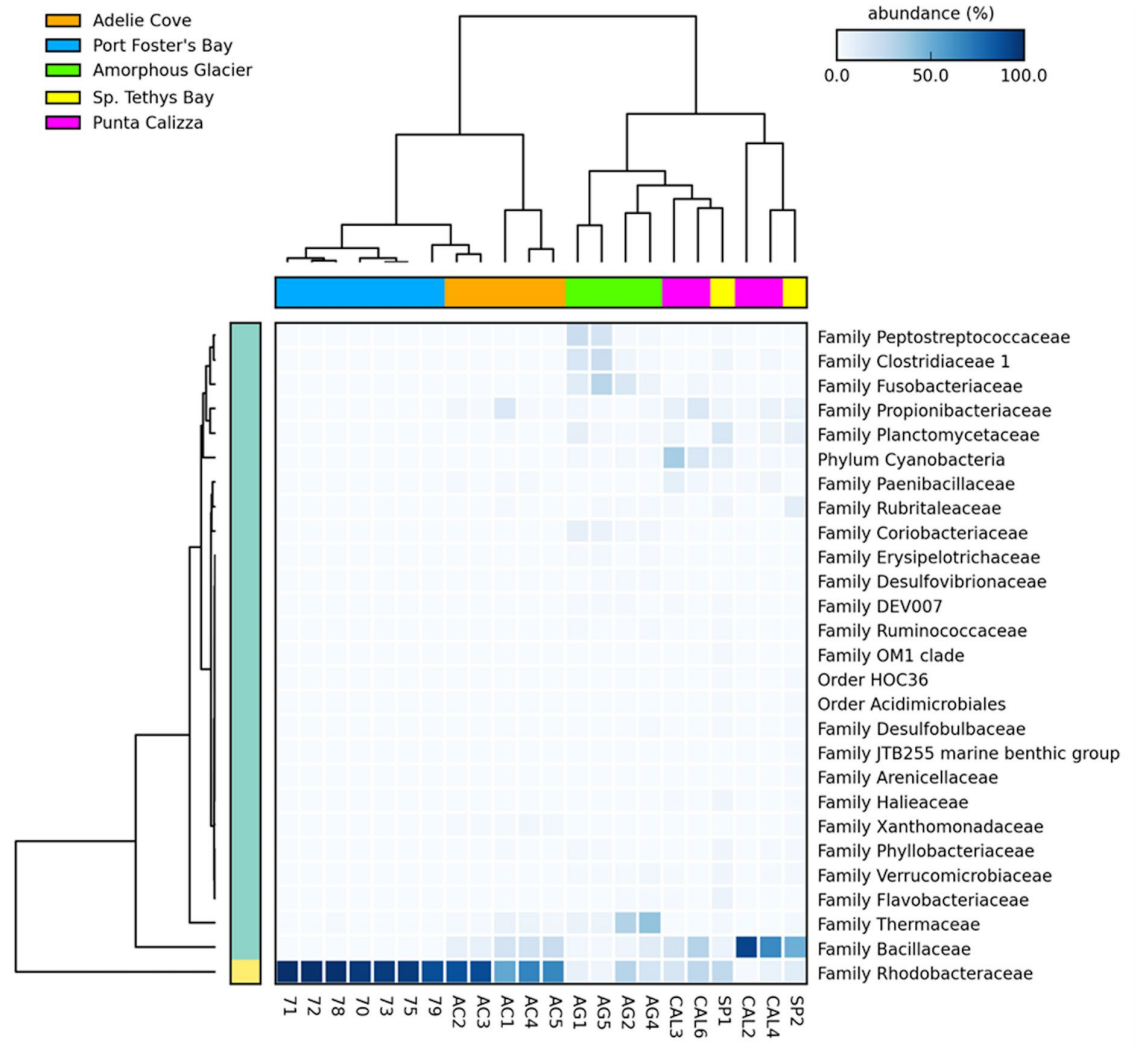
Heatmap showing the relative abundances of the main microbial taxa of individuals of *Odontaster validus* collected in the different Antarctic sites (Statistical test: ANOVA; *Post-hoc* test: Welch’s; value of *p* < 0.05).

Microbiomes of *O. validus* collected within each Antarctic site showed a similarity in the taxonomic composition of 67%, 62%, 56%, 57%, and 65% in Port Foster Bay, Amorphous Glacier, Punta Calizza, Sp. Tethys Bay and Adelie Cove, respectively. Significant differences were found in the microbiomes of *O. validus* comparing the different Antarctic sites (Supplementary Tables 3, 4). Microbiomes of *O. validus* collected in the four sites of the Ross Sea area showed a dissimilarity from 52 to 68% mainly due to the higher abundances of Rhodobacteraceae family in individuals of Adelie Cove than the others (71% vs. 15%, respectively) and to variable contribution of Bacillaceae family (5%, 50%, 27%, 14 in Amorphous Glacier, Punta Calizza, Sp. Tethys Bay and Adelie Cove, respectively). Moreover, bacteria of Thermaceae, Fusobacteriaceae, and Coriobacteriaceae families, observed in individuals of Amorphous Glacier with an average abundance of 20%, 15%, and 5%, were below 3, 0.6% and were totally absent in the other sites, respectively; while bacteria of Planctomycetaceae and Rubritaleaceae observed in individuals of Sp. Tethys Bay with an average abundance of 11% and 6% were below the 3% and 0.5% in the other sites, respectively (Figures 3B, 4). Microbiomes of individuals of Port Foster’s Bay and individuals collected in the sites within the Ross Sea area displayed a dissimilarity in the taxonomic composition from 78 to 81%, except for the microbiomes of individuals of Adelie Cove. Interestingly, the lowest dissimilarity (50%) in microbiomes was found between Port Foster’s Bay (Antarctic Peninsula) and Adelie Cove (Ross Sea; Figure 2). Moreover, 11, 25, 7, 8 and 18 bacterial families were found exclusively in the individuals collected at Port Foster’s Bay, Amorphous Glacier, Punta Calizza, Sp. Tethys Bay and Adelie Cove, respectively.

### 3.3. Putative functions of microbiomes associated with *Odontaster validus*

**Figure 5:**
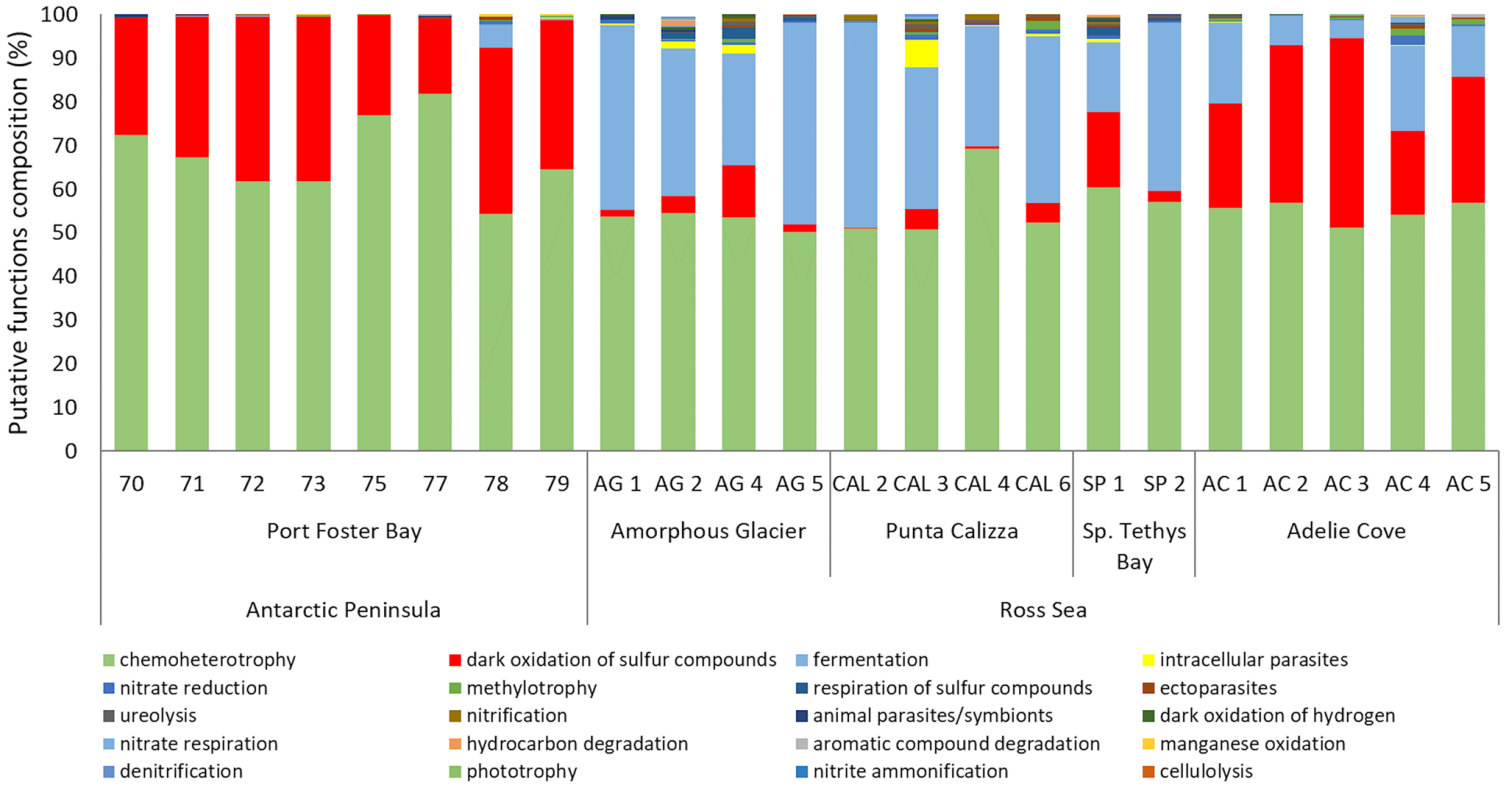
Relative abundance composition of the predicted functions related to microbiomes associated with individuals of *Odontaster validus* collected in the different sites of the Antarctic Peninsula and Ross Sea areas.

Fermentation and oxidation of sulfur compounds were the most represented putative functions in all the individuals of *O. validus*, with values ranging from 50 to 82%, and from 0.3 to 43%, respectively. High percentages of bacteria involved in fermentation were found in all samples with a variable contribution, ranging from 0.1% to 5% in the Antarctic Peninsula individuals and from 4 to 47% in the Ross Sea individuals (Figure 5). Significant differences were found in the putative functions of microbiomes between individuals of Port Foster’s Bay and the other sites, displaying a mean dissimilarity of 40%, except for the microbiomes of *O. validus* collected at Adelie Cove, where the dissimilarity decreased to 22% (Supplementary Figure 5; Supplementary Table 5). Higher percentages of bacteria potentially involved in the oxidation of sulfur compounds were present in Port Foster’s Bay and Adelie Cove (on average 30%) than in the other sites (on average 6%). On the contrary, lower percentages of bacteria involved in fermentation were observed in Port Foster’s Bay and Adelie Cove with an average contribution of 1 and 12% respectively, with respect to the other locations, where it increased up to 33% (Figure 5). Finally, very low percentages (on average < 1%) of parasitic bacteria were putatively ascribed, only in some individuals of Punta Calizza and Amorphous Glacier.

### 3.4. Comparison between microbiomes of *Odontaster validus* and those of the surrounding sediments

**Figure 6:**
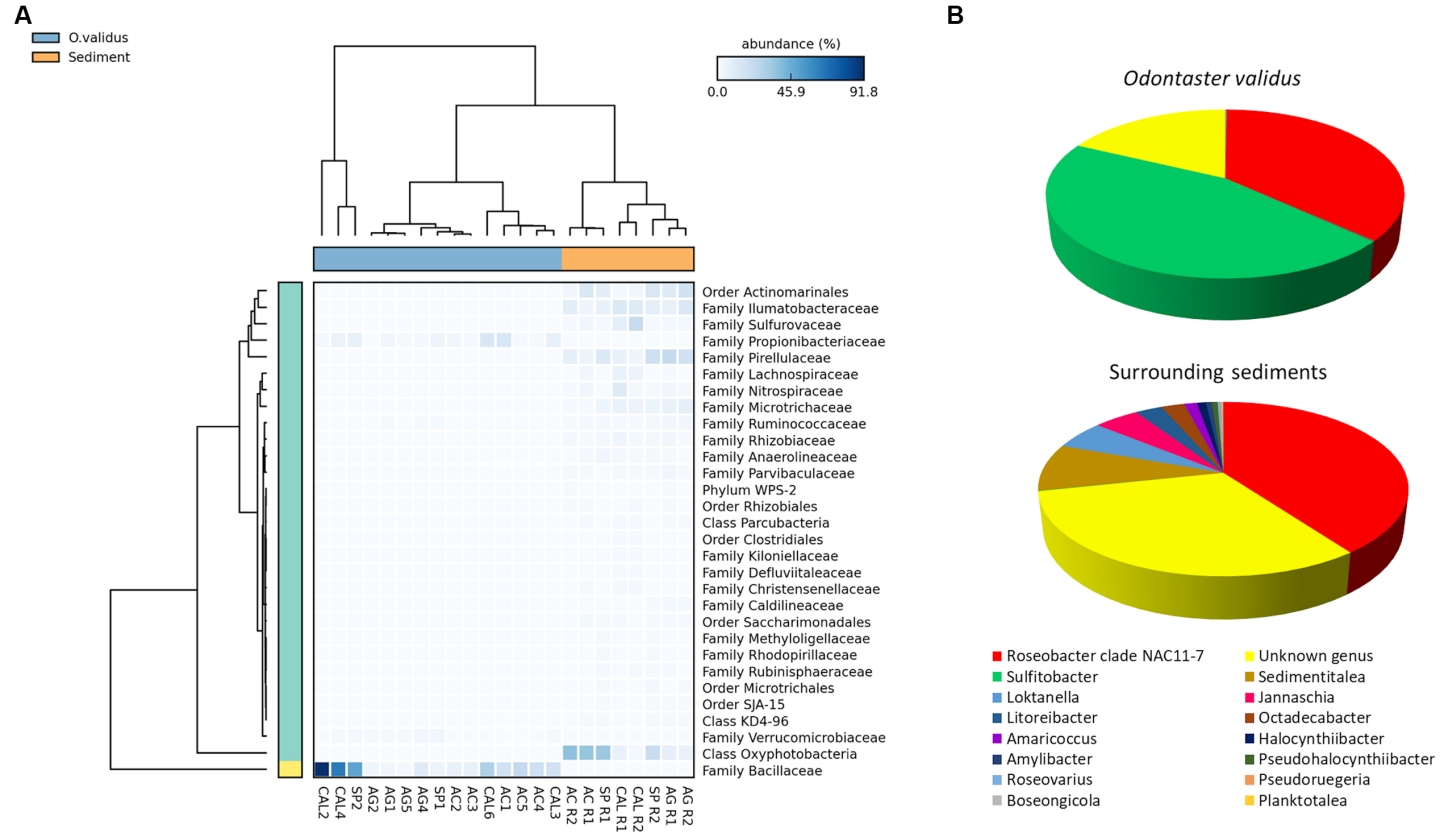
Heatmap showing the relative abundances of the main microbial taxa responsible for the significant differences between individuals of *Odontaster validus* and surrounding sediments (Statistical test: ANOVA; *Post-hoc* test: Welch’s; value of *p* < 0.05) **(A)**. Contribution of all the bacterial genera within the Rhodobacteraceae family detected in the microbiomes associated with *O. validus* and in those living in the surrounding sediments **(B)**.

No significant differences were found in ASV richness either between microbiomes associated with individuals of *O. validus* and living in surrounding sediments, or among microbiomes of sediments collected in different sites (Supplementary Figure 6A). However, significant differences were found between microbiomes associated with *O. validus* and with surrounding sediments in terms of β-diversity (Supplementary Figure 6B) and taxonomic composition (Supplementary Tables 3, 4). Sixteen different bacterial phyla were identified, among which α-Proteobacteria, Acidobacteria, Cyanobacteria, Planctomycetes and Firmicutes were the most quantitatively relevant (average contribution of 25%, 24%, 21%, 14%, and 5%, respectively). Moreover, among the 25 bacterial families identified only the family of Rhodobacteraceae was shared among all samples of sediments and sea stars, showing an average contribution of 33% and 21% in the sea stars and sediments, respectively (Supplementary Figure 7). However, within the Rhodobacteraceae family, only the *Roseobacter* genus was present in both groups, with an average contribution of 40%. The genus *Sulfitobacter*, present in the sea stars with a contribution of 45%, was absent in the sediments. Vice versa, several different genera of the Rhodobacteraceae family were exclusively found in the sediments (Figure 6B). Dissimilarities between sea stars and sediments were mostly driven by the Bacillaceae family, present only in the sea stars with an average contribution of 23%, and by the Pirellulaceae family and the Oxyphotobacteria class, present only in the sediments with an average contribution of 12% and 19%, respectively (Figure 6A). Exclusive bacterial taxa were found both in the sea stars and in the sediments, accounting for 127 and 70, respectively.

## 4. Discussion

### 4.1. Diversity and functions of the bacterial microbiome of *Odontaster validus* from different Antarctic sites

Different studies investigating the relationship between bacteria and sea stars have highlighted the key role of microbiota in the well-being and metabolism of the host (Lawrence et al., 2010; Galac et al., 2016; Carrier and Reitzel, 2017), in increasing the adaptation of the host to the environmental changes (Carrier et al., 2018; Høj et al., 2018; Jackson et al., 2018) and in influencing the host physiology and health status (González-Aravena et al., 2016; Aquino et al., 2021; Loudon et al., 2023). The results of our study highlighted significant differences in the microbiomes of individuals of *O. validus* among the different sampling Antarctic sites. In Port Foster’s Bay (Antarctic Peninsula) microbiomes were characterized by a low bacterial richness (as ASV number) and a taxonomic composition dominated by three ASVs belonging to the Rhodobacteraceae family, which contributed up to 99% to the total composition. Conversely, individuals of *O. validus* collected in the four sites of the Ross Sea were characterized by microbiomes with a high variability both in terms of ASVs richness and taxonomic composition, with three core families (Rhodobacteraceae, Bacillaceae, and Propionibacteriaceae families) shared in all the individuals, and diversified core microbiomes specific for each site.

Nevertheless, the *Rhodobacteracea* family, mainly represented by the *Sulfitobacter* and *Roseobacter* genera, was found in all the individuals of *O. validus* and was also the major driver of relative microbial dissimilarities, between Antarctic zones, and across the Ross Sea sites. Bacteria belonging to these genera were previously found associated with corals, anemones, and sea stars and have been reported to be involved in the carbon cycling and in the degradation of aromatic and sulfur compounds, with important consequences for the nutrition and health of hosts (Ivanova et al., 2004; Raina et al., 2008; Du et al., 2010; Pujalte et al., 2014). A key role in the life of the host could also be played by the bacteria members of the Bacillaceae and Propionibacteriaceae families, which have been found in associations with Antarctic sponges, sea urchins and corals and have shown antimicrobial and antifungal abilities; their presence can promote microbiome stabilization, avoiding infections by opportunists and potential pathogens (Lo Giudice and Rizzo, 2018).

A dominance of Alphaproteobacteria, and the presence of Actinobacteria and Firmicutes, as found in our study, seems to be regular pattern described in sea star microbiomes, including the species *O. validus* (Høj et al., 2018; Jackson et al., 2018; Núñez-Pons et al., 2018, considering the healthy individuals). Therefore, our results expand the available information and suggest a common and stable association between these bacterial groups and sea stars.

Relevant contributions of bacteria belonging to Fusobacteriaceae and Planctomycetaceae were found in some individuals of *O. validus* collected in the Ross Sea area; these bacteria have been already found in association with other Antarctic marine organisms (Webster and Bourne, 2007; Kim et al., 2020; Rodríguez-Barreras et al., 2023) and considered key players in global carbon and nitrogen cycles (Olsen, 2014; Dedysh and Ivanova, 2019). Interestingly, our results indicated the presence of bacteria belonging to the Thermaceae family in some of our individuals of *O. validus*. The presence of such bacteria which have been found especially in terrestrial and marine environments characterized by high temperature regimes (Albuquerque and da Costa, 2014) suggests a high tolerance even to low temperatures and a role in the sea star adaptation to Antarctic conditions.

Our results highlighted a high dissimilarity between microbiomes from the two different geographical sectors (50%–81%; Antarctic Peninsula vs. Ross Sea). However, we also found a high dissimilarity among microbiomes of individuals of *O. validus* collected within the same geographical area (i.e., in the Ross Sea; 52%–68%). In both cases, the high dissimilarity observed was mainly explained by the different contributions of bacteria of the Rhodobacteraceae family.

Geographic location has been recognized among the main drivers of microbiomes’ diversity in many investigations on marine organisms (Pantos et al., 2015; Rubio-Portillo et al., 2018; van de Water et al., 2018; Griffiths et al., 2019). Our results allow us to hypothesize that besides effects due to geographic location (i.e., Antarctic Peninsula vs. Ross Sea and among sites within the Ross Sea area) in influencing the intra-specific composition of microbiomes, other factors, including the biological ones (e.g., health status, feeding behavior) can have a relevant role (Carrier et al., 2018; Høj et al., 2018; Núñez-Pons et al., 2018).

From the analysis of putative functions of the investigated microbiomes, we found that a relevant contribution (more than 50%) of bacterial taxa associated with all individuals of *O. validus* was involved in chemoheterotrophy, despite other different putative functions were identified. This resulted in relatively low dissimilarity values not only among microbiomes of different sites within the Ross Sea (18-32%) but also between microbiomes of the two different geographic sectors (23%–44%).

Collectively, these results suggest an uncoupling between the relatively high dissimilarity of the taxonomic composition of the *O. validus* microbiomes and the relatively low dissimilarity of their putative functions. Such a low dissimilarity of putative functions of microbiomes leads to hypothesize a high functional redundancy of different microbial taxa associated with the *O. validus* likely needed for coping with the extreme Antarctic conditions (Louca et al., 2018).

### 4.2. Origin of the bacterial microbiome associated with *Odontaster validus* in the Ross Sea

The origin of bacteria living in associations with their hosts is largely unknown for most species of marine organisms. Stable bacterial members can be vertically transmitted through generations and can constitute the main fraction of the microbiomes (Bright and Bulgheresi, 2010; Kwan et al., 2017). However, environmental factors and/or biological features (i.e., feeding strategy, health status, metabolic state) can be also responsible for the selection of specific bacteria by the host during its life (Cleary et al., 2019; Britstein et al., 2020). Our investigation revealed a high dissimilarity between the microbiomes associated with the sea stars and those inhabiting the surrounding sediments. Among all the bacterial taxa found, only one was shared between the two groups, the *Roseobacter* genus (family Rhodobacteraceae). Members of this genus have been found also associated with invertebrates (Buchan et al., 2005; Raina et al., 2008; Morrow et al., 2018; Ruocco et al., 2021). The higher percentages of *Roseobacter* in the sea star individuals than in the sediments could be due to a selection process by the sea star species *O. validus* during which these benthic bacteria may have found an ideal niche in the host tissues, thus creating a stable association useful for holobiont life (Adair and Douglas, 2017; Jackson et al., 2018; Koskella and Bergelson, 2020). Moreover, the exclusive occurrence of several bacterial families in *O. validus* (i.e., undetected in the sediments), suggests potential vertical transmission through host selection of these bacterial taxa by the ancestor, or their current acquisition through predator activity (Carrier et al., 2018; Chiarello et al., 2018; Escalas et al., 2021).

## 5. Conclusion

This investigation provides new insights into the knowledge of the microbiomes associated with Antarctic invertebrates, expanding information on diversity, functions, and origin of bacterial taxa belonging to the microbiomes. Although a bacterial core was observed in the *O. validus* specimens investigated (mostly represented by the family Rhodobacteraceae), the richness and taxonomic composition of the microbiomes significantly changed among different Antarctic sectors and within a single area (e.g., Ross Sea). This suggests that besides the geographic sector, other environmental and/or biological factors may influence the microbiome composition of these Antarctic invertebrates. Members belonging to the bacterial core (including Propionaceae and Bacillaceae in addition to Rhodobacteraceae) may play a fundamental role in the sea stars’ wellbeing, potentially establishing commensalism and symbiotic relationships with their hosts and contributing to the metabolic pathways of a wide array of inorganic and organic compounds. Since bacteria belonging to the *Roseobacter* genus were found not only in all specimens of *O. validus* but also in their surrounding sediments, we hypothesize a selection mechanism of the host from the environment to acquire these key holobiont members.

## Data availability statement

The data presented in the study are deposited in the SRA of NCBI repository, BioProject: PRJNA984618, accession numbers: SRR24962722 to SRR24962749, and SRR24949814 to SRR24949821.

## Author contributions

CC and AD conceived the study. ML, LN-P, and CA collected the samples. EB conducted the analysis and wrote the first draft of the manuscript. MT and SS supported EB in bioinformatic and phylogenetic analysis. EB, AD, MT, SS, ML, LN-P, CA, and CC participated in the writing and revision of the manuscript. All authors contributed to the article and approved the submitted version.

## Funding

This study has been conducted in the framework of the project PNRA16_00173 “Diversity and Evolution of Marine Microbial Communities associated with Antarctic Benthic Invertebrates (DEMBAI)” and in the framework of the ACTIQUIM-2 project (CTM2010-17415; cruise ACTIQUIM-4).

## Conflict of interest

The authors declare that the research was conducted in the absence of any commercial or financial relationships that could be construed as a potential conflict of interest.

## Publisher’s note

All claims expressed in this article are solely those of the authors and do not necessarily represent those of their affiliated organizations, or those of the publisher, the editors and the reviewers. Any product that may be evaluated in this article, or claim that may be made by its manufacturer, is not guaranteed or endorsed by the publisher.

## Supplementary material

The Supplementary material for this article can be found online at: https://www.frontiersin.org/articles/10.3389/fmicb.2023.1234725/full#supplementary-material
