# Supplementary material for: Rhodobacteraceae dominate the core microbiome of the sea star Odontaster validus (Koehler, 1906) in two opposite geographical sectors of the Antarctic Ocean
Source: Front Microbiol. 2023 Sep 20;14:1234725. doi: 10.3389/fmicb.2023.1234725 (PMC10548270; doi:10.3389/fmicb.2023.1234725)
Supplement: Supplementary file 1 [file Data_Sheet_1.docx]

Supplementary Material

Rhodobacteraceae dominate the core microbiome of the sea star *Odontaster validus* (Koehler, 1906) from two opposite geographical sectors of the Antarctic Ocean

Emanuela Buschi, Antonio Dell’Anno, Michael Tangherlini, Sergio Stefanni, Marco Lo Martire, Laura Núñez-Pons, Conxita Avila, Cinzia Corinaldesi^*^

*** Correspondence:** Cinzia Corinaldesi: [c.corinaldesi@univpm.it](mailto:c.corinaldesi@univpm.it)

# Supplementary Figures and Tables

## Supplementary Figures


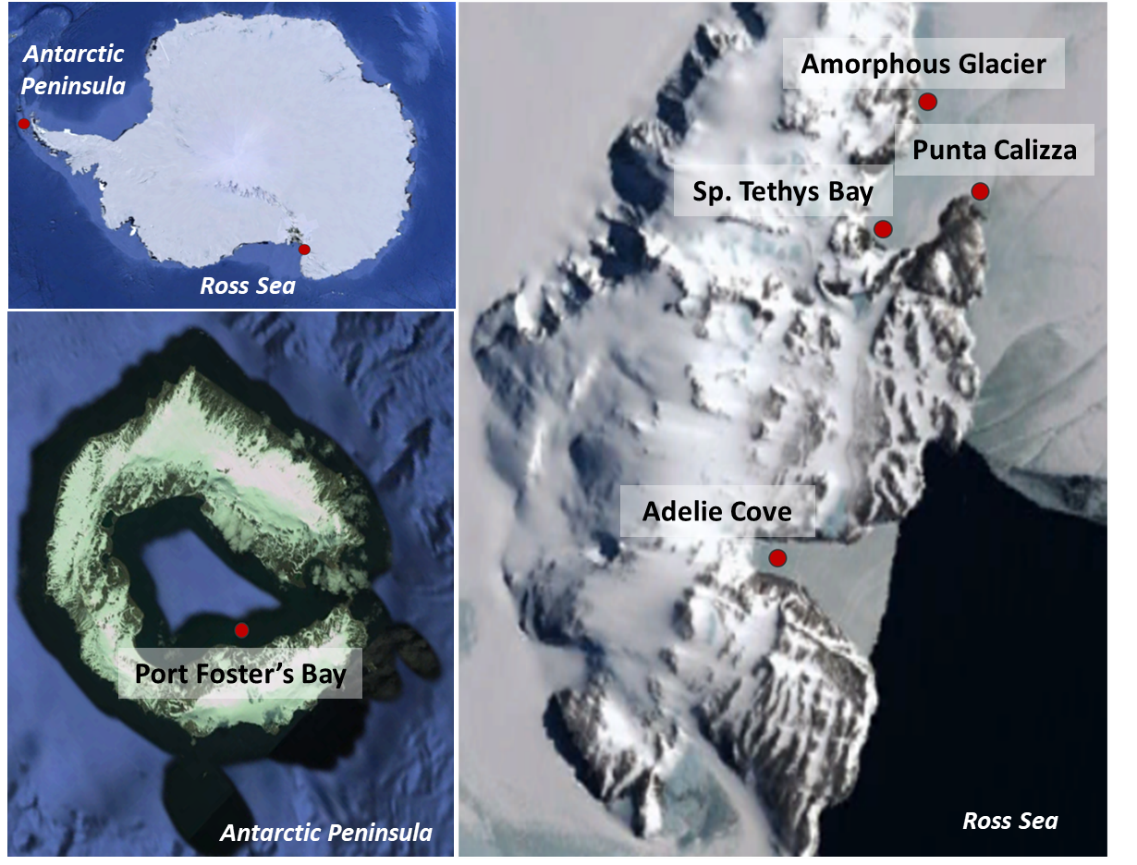


**Supplementary Figure 1.** Map of the different sampling sites in the Antarctic Peninsula and Ross Sea areas where individuals of *O. validus* were collected.


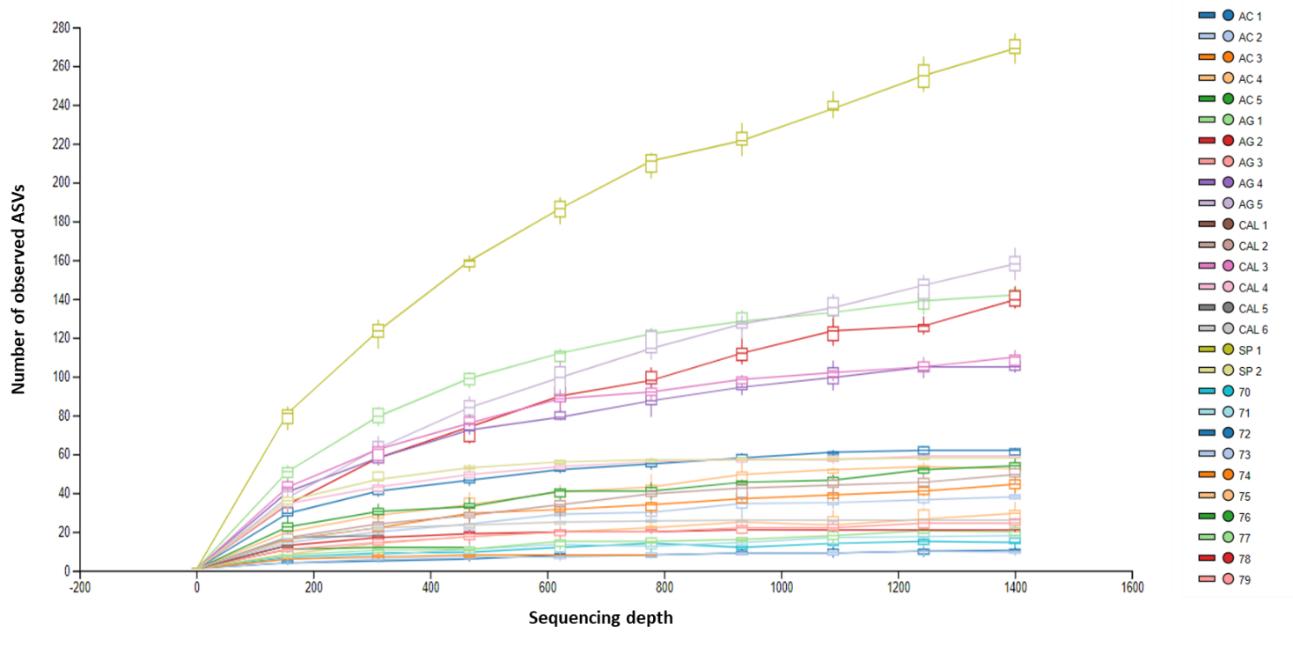


**Supplementary Figure 2.** Rarefaction curve of microbiomes associated with individuals of *O. validus* after a normalization of 1400 sequences for each sample.


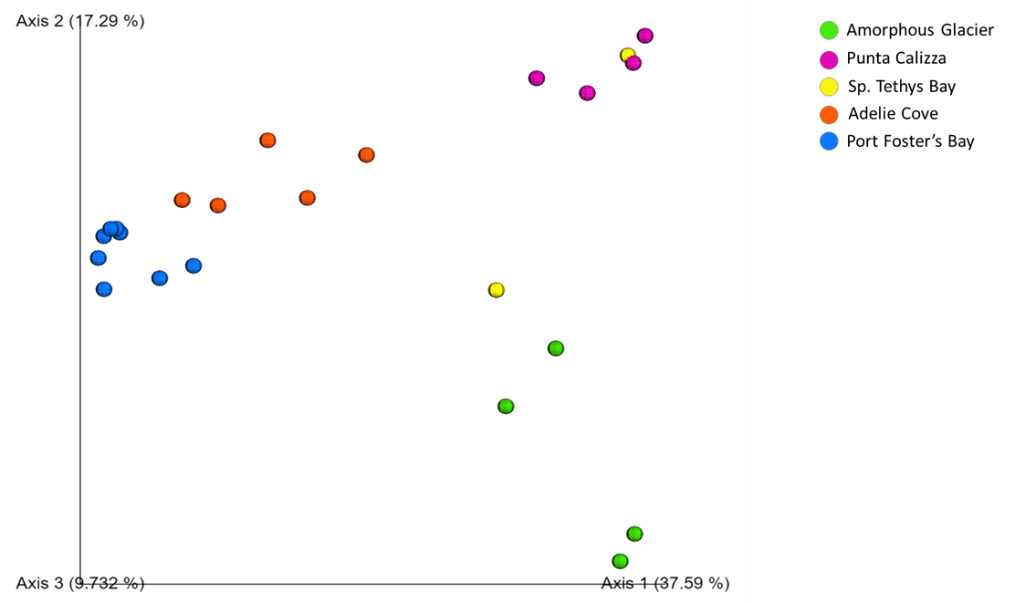


**Supplementary Figure 3.** PCoA plot of microbiomes associated with individuals of *O. validus* collected in the different Antarctic sites of Antarctic Peninsula and Ross Sea areas obtained through the Bray Curtis distance matrix.


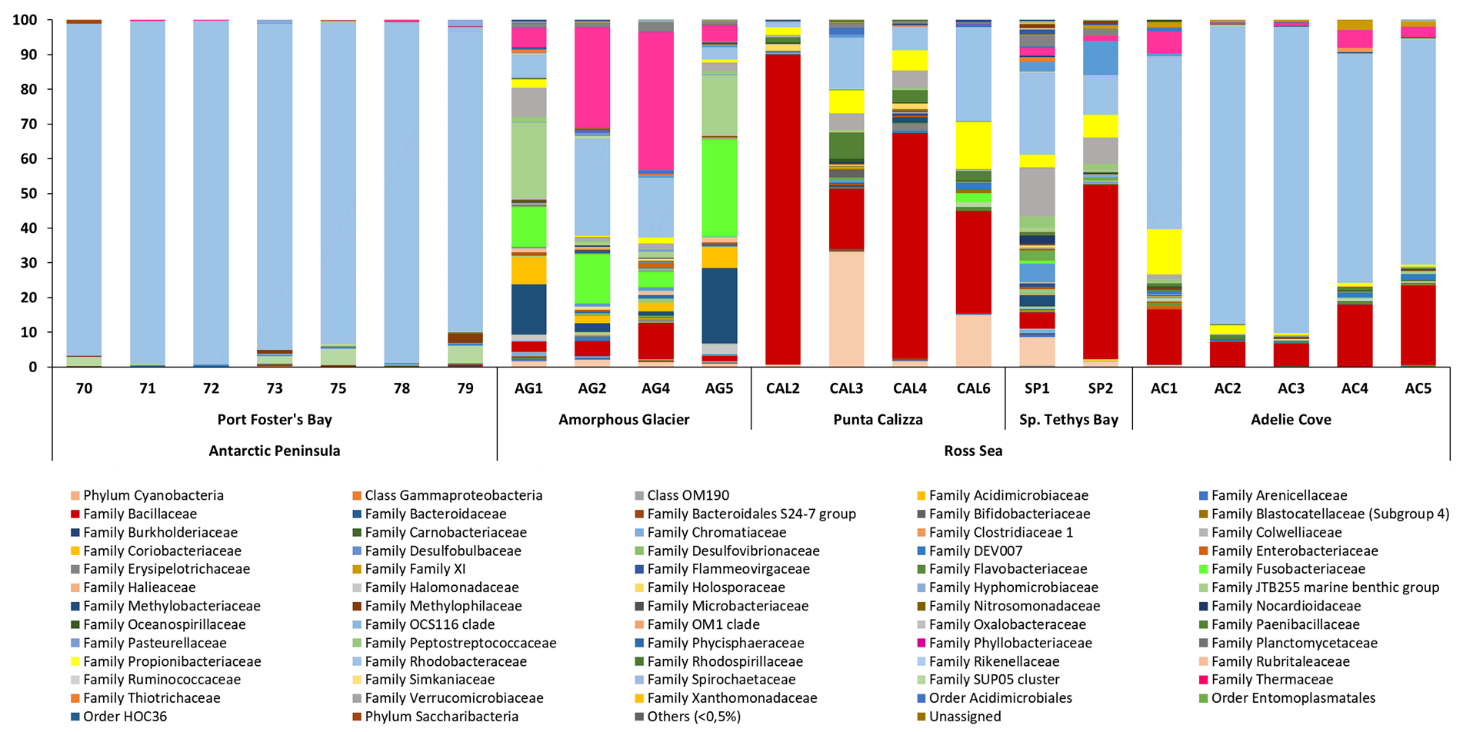


**Supplementary Figure 4.** Sequences contribution (%) of different bacterial taxa (at family level when possible) associated with the *O. validus* individuals in the different Antarctic sites of Antarctic Peninsula and Ross Sea areas. In *Others* all the bacterial taxa with a relative abundance less than 0.5% were grouped.


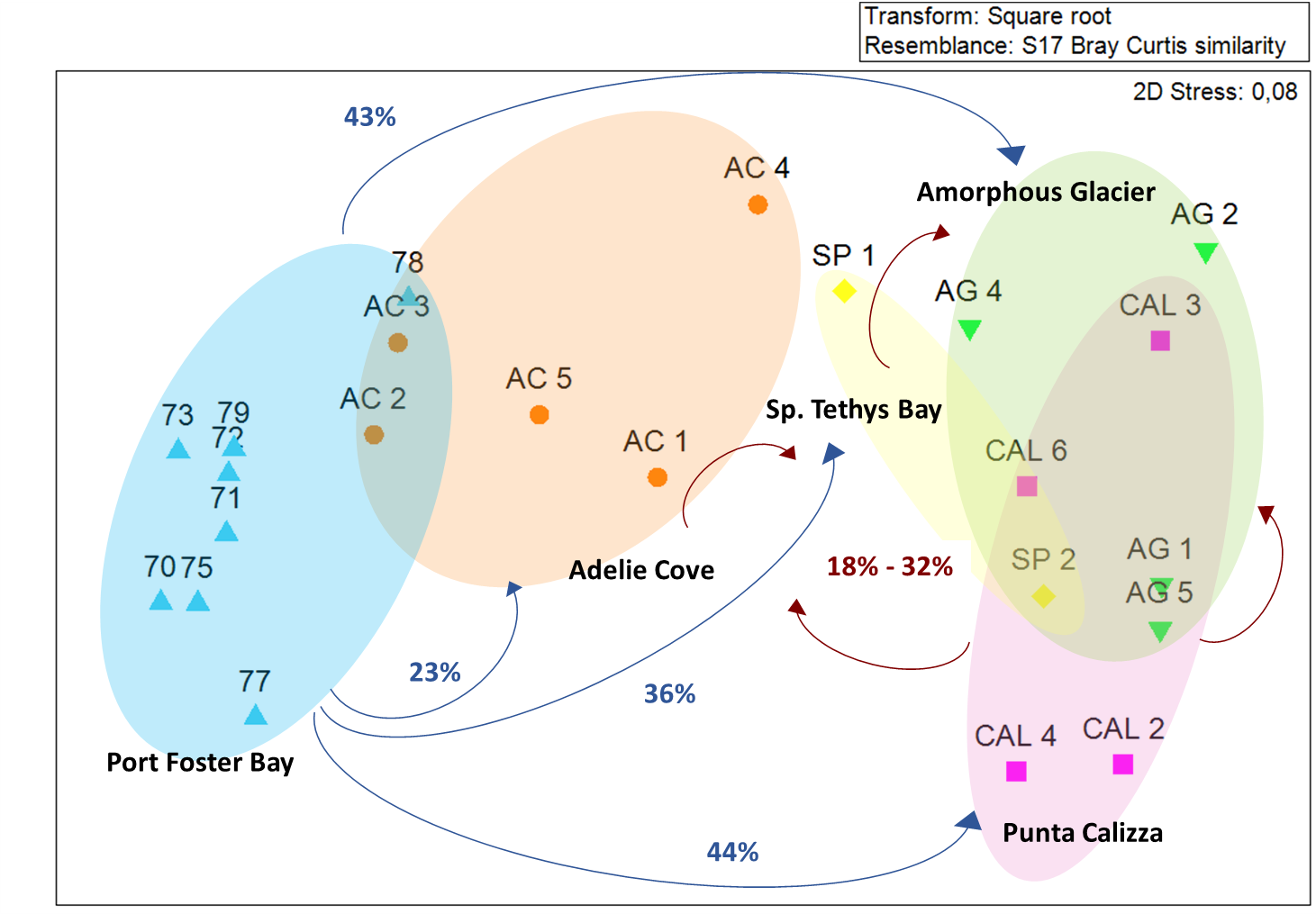


**Supplementary Figure 5.** MDS analysis comparing the putative function composition of microbiomes associated with individuals of *O. validus* collected in the different Antarctic sites of Antarctic Peninsula and Ross Sea areas. Percentages in blue refer to the dissimilarities between Port Foster Bay and the different Ross Sea sites; percentages in red refer to the dissimilarities among the sites within the Ross Sea area.


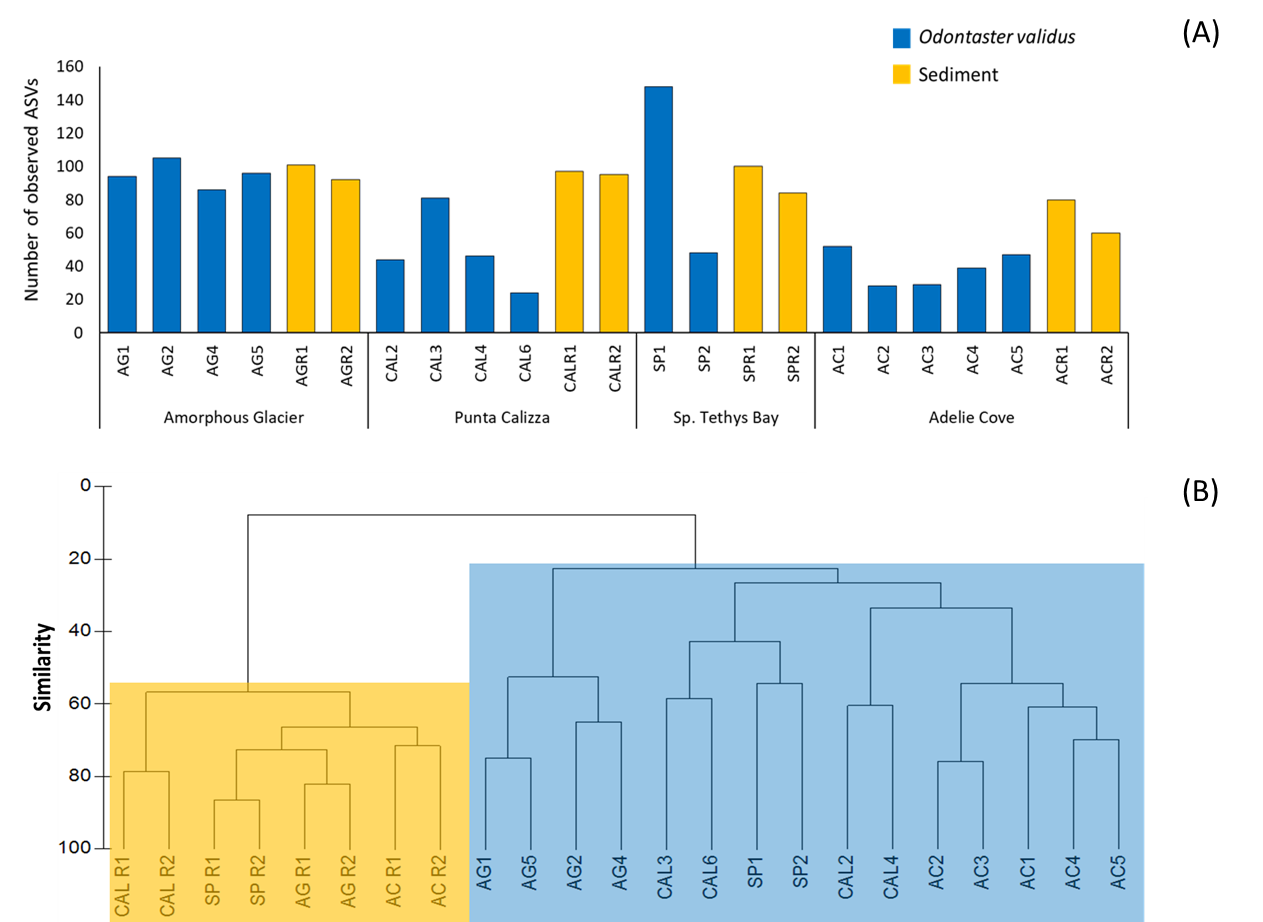


**Supplementary Figure 6**. Alpha- diversity calculated in terms of observed ASVs (A) and Beta-diversity as results of cluster analysis on Bray Curtis dissimilarity (B) in microbiomes associated with *O. validus* and living in surrounding sediments, collected in the four sites of Ross Sea area.


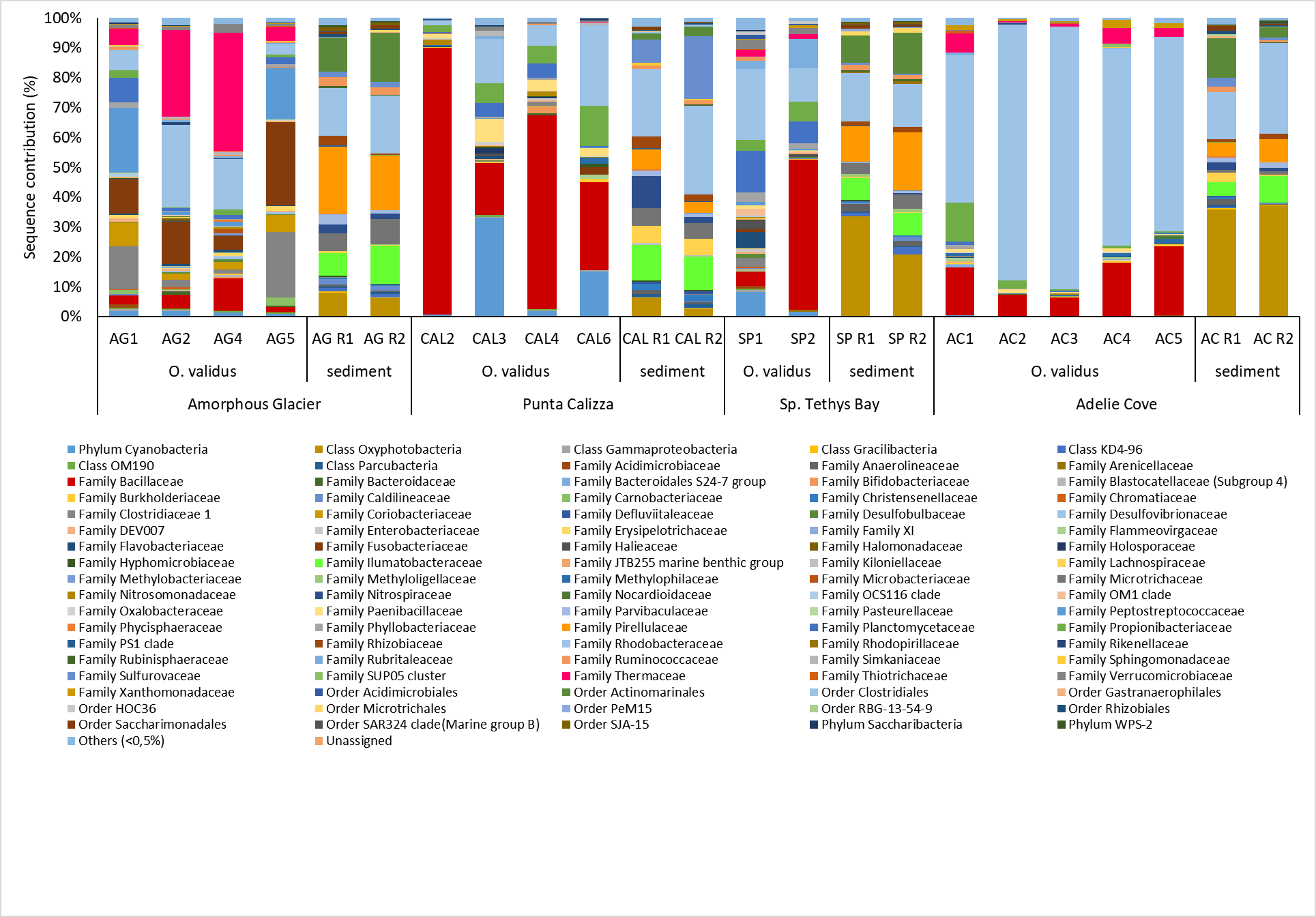


**Supplementary Figure 7**. Sequences contribution (%) of different bacterial taxa (at family level when possible) associated with the *O. validus* individuals and living in the surrounding sediments in the different Antarctic sites within Ross Sea area. In *Others* all the bacterial taxa with a relative abundance less than 0.5% were grouped.

## Supplementary Tables

**Supplementary Table 1.** Table listing the sampling sites organized by geographic location, sites, and geographic coordinates.

| **Geographic location** | **Sites** | **Latitude** | **Longitude** |
| --- | --- | --- | --- |
| Antarctic Peninsula | Port Foster’s Bay | 62° 58' 22.19" S | 60° 38' 59.99" W |
| Ross Sea | Amorphous Glacier | 74°41,237’ S | 164°02,183’ E |
|  | Punta Calizza | 74°40,545’ S | 164°04,095’ E |
|  | Spiaggetta Tethys Bay | 74°42.068’ S | 164°02,514’ E |
|  | Adelie Cove | 74°46,467’ S | 164°00,266’ E |

**Supplementary Table 2.** List of individuals of *O. validus* selected for this study.

| **Geographic location** | **Sites** | **Number of individuals** | **Individuals** |
| --- | --- | --- | --- |
| Antarctic Peninsula | Port Foster’s Bay | 10 | 70, 71, 72, 73, 74, 75, 76, 77, 78, 79 |
| Ross Sea | Amorphous Glacier | 5 | AG1, AG2, AG3, AG4, AG5 |
|  | Punta Calizza | 6 | CAL1, CAL2, CAL3, CAL4, CAL5, CAL6 |
|  | Spiaggetta Tethys Bay | 2 | SP1, SP2 |
|  | Adelie Cove | 5 | AC1, AC2, AC3, AC4, AC5 |

**Supplementary Table 3.** Results of PERMANOVA main test carried out on the microbiomes associated with *O. validus* collected in different Antarctic sites.

| *ASV richness of microbiomes between Antarctic Peninsula and Ross Sea areas* | | | | |
| --- | --- | --- | --- | --- |
|  | Df | SS | F | P |
| Sector | 2 | 1819.2 | 17968 | 0.001 |
| Residual | 20 | 1012.5 |  |  |
| *ASV richness of microbiomes among the different Antarctic sites* | | | | |
|  | Df | SS | F | P |
| Site | 4 | 2135.8 | 13813 | 0.001 |
| Residual | 18 | 695.83 |  |  |
| *Taxonomic composition between Antarctic Peninsula and Ross Sea areas* | | | | |
|  | Df | SS | F | P |
| Sector | 2 | 17779 | 68989 | 0.001 |
| Residual | 20 | 25771 |  |  |
| *Taxonomic composition among the different Antarctic sites* | | | | |
|  | Df | SS | F | P |
| Site | 4 | 30348 | 10344 | 0.001 |
| Residual | 18 | 13203 |  |  |
| *Putative functions of microbiomes between Antarctic Peninsula and Ross Sea areas* | | | | |
|  | Df | SS | F | P |
| Sector | 2 | 5206.9 | 20655 | 0.001 |
| Residual | 20 | 5294 |  |  |
| *Putative functions of microbiomes among the different Antarctic sites* | | | | |
|  | Df | SS | F | P |
| Site | 4 | 7742.2 | 12629 | 0.001 |
| Residual | 18 | 2758.7 |  |  |
| *ASV richness of microbiomes between sea stars and sediments* | | | | |
|  | Df | SS | F | P |
| Source | 1 | 21952 | 18.331 | 0.139 |
| Residual | 21 | 23492 |  |  |
| *Taxonomic composition of microbiomes between sea stars and sediments* | | | | |
|  | Df | SS | F | P |
| Source | 1 | 26866 | 20.594 | 0.001 |
| Residual | 21 | 27396 |  |  |

**Supplementary Table 4.** Results of Simper analysis carried out on the taxonomic composition of microbiomes associated with *O. validus* collected in different Antarctic sites and the main bacterial taxa responsible for the dissimilarities.

| *Simper analysis carried out between Antarctic Peninsula and Ross Sea areas* | | |
| --- | --- | --- |
| Group Antarctic Peninsula | Average similarity: 66.61 | |
| Group Ross Sea | Average similarity: 44.58 | |
| Groups Antarctic Peninsula *vs* Ross Sea | Average dissimilarity = 70.55 | |
| Main responsible bacterial species: | Av.Abund Group 1 | Av.Abund Group 2 |
| Family Bacillaceae | 0.27 | 2.68 |
| Family Rhodobacteraceae | 4.54 | 3.06 |
| Family Propionibacteriaceae | 0.27 | 1.34 |
| *Simper analysis carried out among the different Antarctic sites* | | |
| Group Port Foster Bay | Average similarity: 66.61 | |
| Group Amorphous Glacer | Average similarity: 61.86 | |
| Group Punta Calizza | Average similarity: 55.81 | |
| Group Sp. Tethys Bay | Average similarity: 57.26 | |
| Group Adelie Cove | Average similarity: 65.25 | |
| Groups Port Foster Bay *vs* Amorphous Glacer | Average dissimilarity = 81.29 | |
| Main responsible bacterial species: | Av. Abund_1 | Av. Abund_2 |
| Family Fusobacteriaceae | 0.00 | 2.58 |
| Family Thermaceae | 0.41 | 2.68 |
| Family Rhodobacteraceae | 4.54 | 2.45 |
| Groups Port Foster Bay *vs* Punta Calizza | Average dissimilarity = 78.16 | |
| Main responsible bacterial species: | Av. Abund_1 | Av. Abund_2 |
| Family Bacillaceae | 0.27 | 3.76 |
| Family Rhodobacteraceae | 4.54 | 2.26 |
| Family Propionibacteriaceae | 0.27 | 1.95 |
| Groups Amorphous Glacer *vs* Punta Calizza | Average dissimilarity = 64.84 | |
| Main responsible bacterial species: | Av. Abund_1 | Av. Abund_2 |
| Family Thermaceae | 2.68 | 0.06 |
| Family Fusobacteriaceae | 2.58 | 0.31 |
| Family Bacillaceae | 1.61 | 3.76 |
| Groups Port Foster Bay *vs* Sp. Tethys Bay | Average dissimilarity = 79.20 | |
| Main responsible bacterial species: | Av. Abund_1 | Av. Abund_2 |
| Family Bacillaceae | 0.27 | 2.83 |
| Family Rhodobacteraceae | 4.54 | 2.85 |
| Family Propionibacteriaceae | 0.27 | 1.79 |
| Groups Amorphous Glacer *vs* Sp. Tethys Bay | Average dissimilarity = 54.53 | |
| Main responsible bacterial species: | Av. Abund_1 | Av. Abund_2 |
| Family Fusobacteriaceae | 2.58 | 0.31 |
| Family Thermaceae | 2.68 | 1.05 |
| Family Coriobacteriaceae | 4.63 | 0.00 |
| Groups Punta Calizza *vs* Sp. Tethys Bay | Average dissimilarity = 53.37 | |
| Main responsible bacterial species: | Av. Abund_1 | Av. Abund_2 |
| Family Planctomycetaceae | 1.09 | 2.42 |
| Family Bacillaceae | 3.76 | 2.83 |
| Family Rubritaleaceae | 0.16 | 1.84 |
| Groups Port Foster Bay vs Adelie Cove | Average dissimilarity = 52.41 | |
| Main responsible bacterial species: | Av. Abund_1 | Av. Abund_2 |
| Family Bacillaceae | 0.27 | 2.61 |
| Family Propionibacteriaceae | 0.27 | 1.10 |
| Family Thermaceae | 0.41 | 1.25 |
| Groups Amorphous Glacer vs Adelie Cove | Average dissimilarity = 66.95 | |
| Main responsible bacterial species: | Av. Abund_1 | Av. Abund_2 |
| Family Fusobacteriaceae | 2.58 | 0.00 |
| Family Coriobacteriaceae | 1.63 | 0.00 |
| Family Thermaceae | 2.68 | 1.25 |
| Groups Punta Calizza *vs* Adelie Cove | Average dissimilarity = 55.37 | |
| Main responsible bacterial species: | Av. Abund_1 | Av. Abund_2 |
| Family Rhodobacteraceae | 2.26 | 4.25 |
| Family Bacillaceae | 3.76 | 2.61 |
| Family Thermaceae | 0.06 | 1.25 |
| Groups Sp. Tethys Bay vs Adelie Cove | Average dissimilarity = 58.81 | |
| Main responsible bacterial species: | Av. Abund_1 | Av. Abund_2 |
| Family Planctomycetaceae | 2.42 | 0.30 |
| Family Rubritaleaceae | 1.84 | 0.19 |
| Family Rhodobacteraceae | 2.85 | 4.25 |

**Supplementary Table 5.** Results of Simper analysis carried out on the putative function composition of microbiomes associated with *O. validus* collected in different Antarctic sites.

| *Simper analysis carried out between Antarctic Peninsula and Ross Sea areas* | |
| --- | --- |
| Group Antarctic Peninsula | Average similarity: 87.04 |
| Group Ross Sea | Average similarity: 75.42 |
| Groups Antarctic Peninsula *vs* Ross Sea | Average dissimilarity: 35.36 |
| *Simper analysis carried out among the different Antarctic sites* | |
| Group Port Foster Bay | Average similarity: 87.04 |
| Group Amorphous Glacer | Average similarity: 80.77 |
| Group Punta Calizza | Average similarity: 81.72 |
| Group Sp. Tethys Bay | Average similarity: 75.51 |
| Group Adelie Cove | Average similarity: 83.40 |
| Groups Port Foster Bay *vs* Amorphous Glacer | Average dissimilarity: 43.35 |
| Groups Port Foster Bay *vs* Punta Calizza | Average dissimilarity: 43.59 |
| Groups Amorphous Glacer *vs* Punta Calizza | Average dissimilarity: 20.98 |
| Groups Port Foster Bay *vs* Sp. Tethys Bay | Average dissimilarity: 36.44 |
| Groups Amorphous Glacer *vs* Sp. Tethys Bay | Average dissimilarity: 18.35 |
| Groups Punta Calizza *vs* Sp. Tethys Bay | Average dissimilarity: 22.29 |
| Groups Port Foster Bay *vs* Adelie Cove | Average dissimilarity: 21.95 |
| Groups Amorphous Glacer *vs* Adelie Cove | Average dissimilarity: 31.63 |
| Groups Punta Calizza *vs* Adelie Cove | Average dissimilarity: 30.53 |
| Groups Sp. Tethys Bay *vs* Adelie Cove | Average dissimilarity: 26.18 |
